# The quantitative parameters based on marrow metabolism derived from synthetic MRI: A pilot study of prognostic value in participants with newly diagnosed multiple myeloma

**DOI:** 10.1002/cam4.7109

**Published:** 2024-03-30

**Authors:** Sha Cui, Yinnan Guo, Weiran Niu, Jianting Li, Wenjin Bian, Wenqi Wu, Wenjia Zhang, Qian Zheng, Jun Wang, Jinliang Niu

**Affiliations:** ^1^ Department of Medical Imaging Shanxi Medical University Taiyuan China; ^2^ Department of Radiology Second Hospital of Shanxi Medical University Taiyuan China; ^3^ Department of Pain Fifth Hospital of Shanxi Medical University Taiyuan China

**Keywords:** early treatment response, magnetic resonance imaging, multiple myeloma, R‐ISS stage, tumor burden

## Abstract

**Background:**

The value of SyMRI‐derived parameters from lumbar marrow for predicting early treatment response and optimizing the risk stratification of the Revised International Staging System (R‐ISS) in participants with multiple myeloma (MM) is unknown.

**Methods:**

We prospectively enrolled participants with newly diagnosed MM before treatment. The SyMRI of lumbar marrow was used to calculate T1, T2, and PD values and the clinical features were collected. All participants were divided into good response (≥VGPR) and poor response (<VGPR) groups after four treatment cycles. Univariate, multivariate analyses and ROC were used to identify prognostic significance. Mann–Whitney *U*‐tests were used to compare the SyMRI parameters between genders. The value of optimizing the risk stratification was analyzed by Fisher's exact tests at each R‐ISS stage.

**Results:**

Fifty‐nine participants (good response, *n* = 33; poor response, *n* = 26) were evaluated. The bone marrow plasma cell percentage, β_2_‐microglobulin, T1 and T2 value were difference between two groups (all *p* < 0.05). The T1 (odds ratio 1.003, *p* = 0.005) and T2 values (odds ratio 0.910, *p* = 0.002) were independent predictors and the AUC and cut‐off values were 0.787, 967.2 ms and 0.784, 75.9 ms, respectively. There were no significant differences in SyMRI parameters between genders. Participants with both T1 value ≥967.2 ms and T2 value ≤75.9 ms in the R‐ISS II stage were potentially to get poor response.

**Conclusions:**

Synthetic MRI is a promising tool for predicting early treatment response to MM and promoting R‐ISS II stage risk stratification.

## 1 INTRODUCTION

Multiple myeloma (MM) is the most common hematologic malignancy in elder population.^1^ The monoclonal plasma cells infiltrate the primary bone marrow (BM) sites from where they further migrate into peripheral blood cells, and then spread and settle at distant BM sites.^2^ The patients with plasma cell proliferative disorders could benefit from accurately assessing their tumor burden to determine the timing and strategy of treatment for improving prognosis.^3^ Currently, beta‐2‐microglobulin and lactate dehydrogenase (LDH) were common factors used to assess MM tumor burden, however, which might be influenced by renal function or glucose metabolism.^4^, ^5^ The Revised International Staging System (R‐ISS), based on these biomarkers of tumor burden and highrisk cytogenetic abnormalities (CA) [deletion (17p), translocation t (4;14), or t (14;16)], is currently the most widely used risk stratification method.^6^, ^7^ However, up to 62% of MM patients are classified as R‐ISS II stage, and their prognosis is highly heterogeneous.^8^

Magnetic resonance imaging (MRI) is sensitive to detect BM infiltration before the occurrence of bone destruction, which is a prior imaging method for evaluating radiological tumor burden of marrow infiltrated disease.^9^ So, the whole‐body MRI (WB‐MRI) was recommended by the International Myeloma Working Group (IMWG) for tumor burden evaluation in newly diagnosed MM patients.^3^ However, WB‐MRI is time‐consuming and unable to be widely used in clinical work. At present, metabolic tumor burden derived from the maximum standardized uptake value (SUV_max_) in fluorodeoxyglucose (FDG) positron emission tomography (PET)/CT potentially provides a better indication for the metabolic tumor burden of MM.^1^ Simultaneously, quantitative parameters obtained by MRI Functional technology were also used to evaluate the metabolic tumor burden of MM, but these parameters could only reflect the abnormality of single tissue component (cellularity, fat, etc.) in infiltrated marrow.^10^, ^11^ So, it is necessary to find a new imaging method to evaluate the total metabolic disorder caused by plasma cells infiltration of the marrow microenvironment.

The T1 and T2 values were MRI biological parameters used to analyze tissue composition.^12^ These parameters would change if pathological processes alter the water composition or local molecular environment of tissues.^12^ Compared with traditional T2 mapping using multi‐echo (ME) technique,^13^ the synthetic MRI (SyMRI) uses multi‐delay multi‐echo (MDME) acquisition technique to simultaneously generate quantitative T1, T2, and proton density (PD) values in one scan within a few minutes.^14^ The parameters had been used to various diseases in the differentiation of benign and malignant lesions, and the evaluation of prognostic factors or posttreatment changes.^15^, ^16^, ^17^ It has previously been proposed that these parameters may comprehensively characterize metabolic changes in BM composition caused by tumor cells proliferation in patients with hematological diseases.^18^ We hypothesized that the parameters of SyMRI may reflect the overall pathological changes of infiltrated marrow in MM, and can be used to evaluate the metabolic tumor burden.

The depth of early treatment response is associated with survival outcomes in MM.^19^ This study tried to use the quantitative parameters derived from SyMRI technique to predict the early treatment response in participants with newly diagnosed MM, and to explore their usefulness in optimizing the R‐ISS stage.

## 2 METHODS

### 2.1 Participant cohort

This prospective study received approval from the ethics committee of the Second Hospital of Shanxi Medical University (protocol number 2020‐040). All participants provided written informed consent for the performance of MRI examination and for the use of clinical, laboratory, and imaging data. Inclusion criteria were participants with newly diagnosed MM who were determined by the IMWG criteria^3^ from August 2020 to July 2022, had not received any treatment, and could tolerate MRI examination. Exclusion criteria were participants who had any other diseases in lumbar marrow, had not completed the 4 cycles of induction chemotherapy in our hospital or inferior quality of MRI images. Clinical data and laboratory test results including gender, age, hemoglobin, platelet, serum albumin, serum β_2_‐microglobulin, serum LDH, serum calcium, bone marrow plasma cell (BMPC) percentage, and flow cytometry of BM cells were collected at the first diagnosis. Participants were staged by the R‐ISS staging system before treatment. Participants were treated with one of the following first‐line induction regimens: bortezomib, lenalidomide, dexamethasone (*n* = 42); bortezomib, dexamethasone (*n* = 9); bortezomib, thalidomide, dexamethasone (*n* = 5); bortezomib, cyclophosphamide, and dexamethasone (*n* = 3). Treatment response was evaluated according to the IMWG response criteria^20^ after the completion of 4 cycles of induction therapy. The treatment response categories include stringent complete remission (sCR), complete response (CR), VGPR, partial response (PR), stable disease (SD), and progressive disease. Participants were categorized into two groups: good response group (sCR, CR, and VGPR status) and poor response group (PR, SD, and progressive disease status).

### 2.2 BMPC percentage calculation

The samples of BM biopsy were obtained from unilateral ilium in all participants at the time of diagnosis. The formalin fixed and paraffin embedded specimens were cut into 4 μm thick sections and subjected to hematoxylin–eosin (HE) and periodic acid schiff (PAS) staining.

Slides with 4 μm thick sections were deparaffinized, hydrated and immunohistochemically stained with anti‐CD138, anti‐CD38, anti‐CD56, MUM1, Ig‐kappa, and Ig‐lambda antibodies. Three fields were selected at 400× magnification with 50 cells in each field. The ratio of immunolabeled plasma cells to nucleated cells in each field was calculated, and the mean value of the three fields was taken as the BMPC percentage.

### 2.3 MRI examination

MRI from all participants in this study were conducted on the 3.0 T MR system (Signa Pioneer, GE Healthcare, Milwaukee, WI). Participants were positioned supine (feet‐first) with a bellyband was added to reduce respiratory artifacts, and a 64‐channel spine coil and a 16‐channel phased‐array body coil were used for the examination of the lumbar spine. The sequences included sagittal fast spin‐echo (FSE) T_1_WI, sagittal iterative decomposition of asymmetric echoes (IDEAL) T_2_WI, and sagittal SyMRI. The SyMRI used a 2D FSE MDME sequence, which contains four automatically calculated saturation delays and two echo times. The scanning parameters of SyMRI were as follows: echo time (TE), 21/95 ms; recovery time (TR), 4000 ms; field of view (FOV), 32 cm; 20 slices of 4 mm thickness and 1 mm interval; image matrix = 320 × 192, bandwidth = 31.25 kHz, acquisition time = 3 min and 28 s.

Two radiologists (J.N. and S.C, with 13 and 7 years of experience in musculoskeletal imaging, respectively) were responsible for the measurement of lumbar SyMRI parameters. When there was disagreement on the results, the two radiologists discuss and reach an agreement.

### 2.4 Measurement of T1, T2, and PD values

The SyMRI‐derived images [T_1_WI, T_2_WI, short‐inversion‐time inversion recovery (STIR), T1 map, T2 map, and PD map] were generated using the MR image compilation (MAGIC, version 100.0.0.) modules of the MRI console. The T1, T2, and PD values were automatically calculated when the area of interest (ROI)s were drawn on the central slice of the synthetic T_2_WI image and then switched to quantitative maps.

**FIGURE 1:**
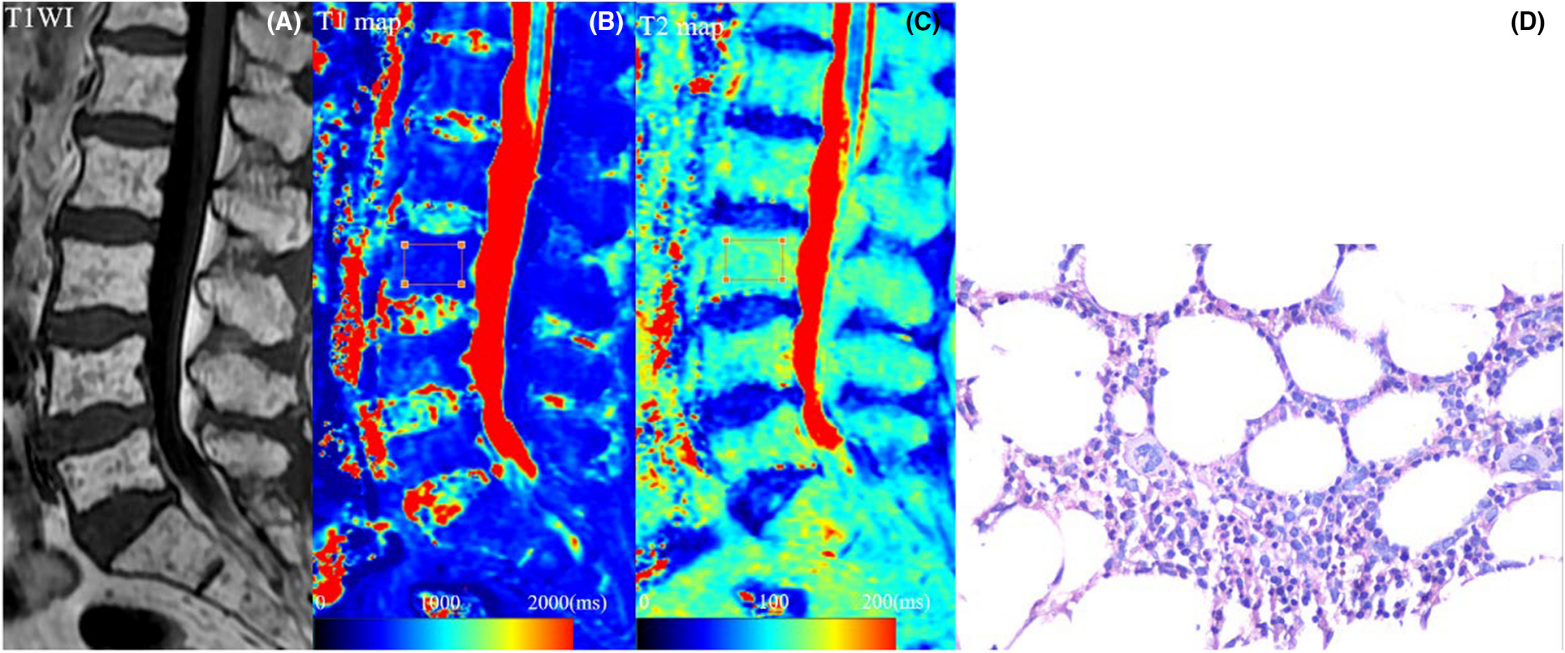
A 63‐year‐old female MM participant with a serum β_2_‐microglobulin level of 1.70 mg/L, achieved CR after 4 cycles of induction therapy. (A) Sagittal T_1_WI image of the lumbar marrow showed a salt‐and‐pepper infiltration pattern. (B, C) On T1 and T2 maps, the ROI was manually drawn on the center region of the vertebral body from L1 to L5, and the mean values of T1 and T2 values were 606.4 and 102.3 ms, respectively. (D) Photomicrograph (400× magnification; HE and PAS stain) showed the BMPC percentage was approximately 15%. BMPC, bone marrow plasma cell; CR, complete response; HE, hematoxylin eosin; MM, multiple myeloma; PAS, periodic acid schiff; ROI, area of interest.

**FIGURE 2:**
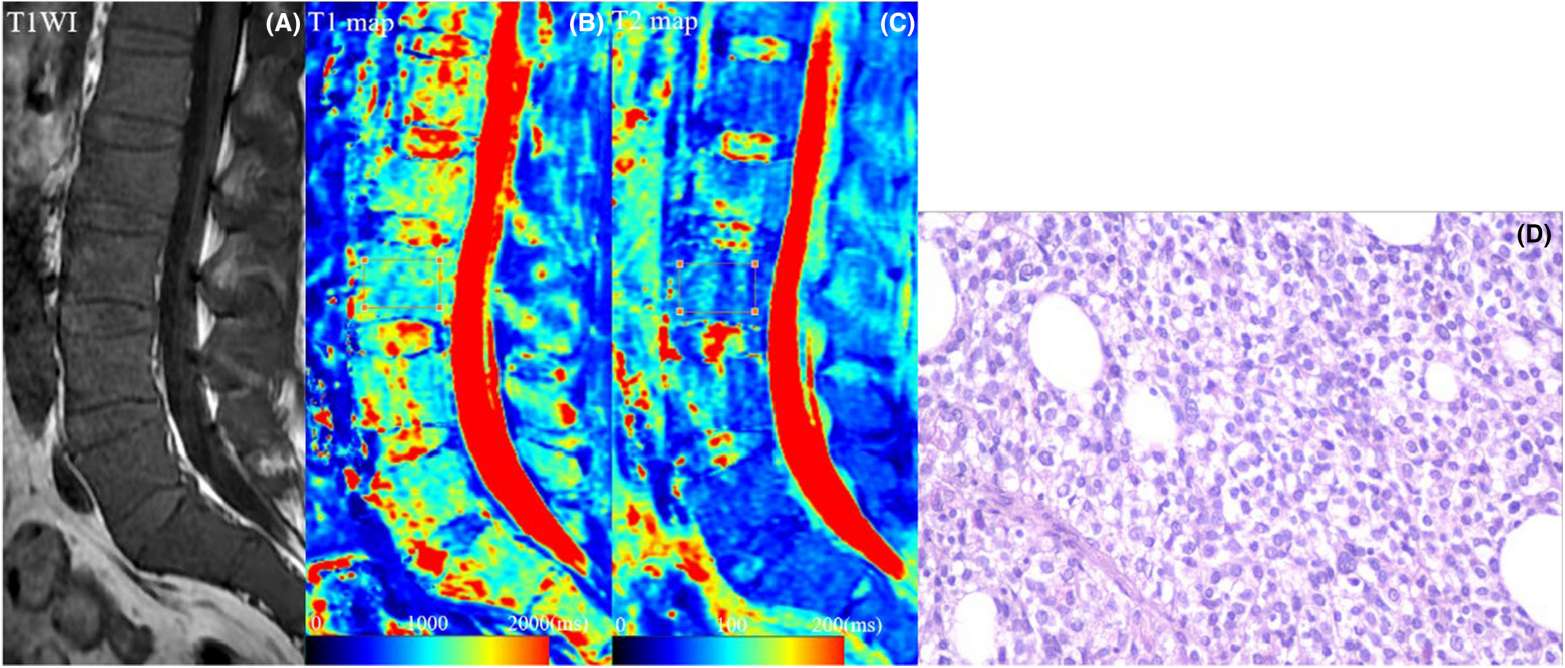
A 50‐year‐old male MM participant with a serum β_2_‐microglobulin level of 9.58 mg/L, achieved PR after 4 cycles of induction therapy. (A) Sagittal T_1_WI image of the lumbar marrow showed a diffuse infiltration pattern. (B, C) On T1 and T2 maps, the ROI was manually drawn on the center region of the vertebral body from L1 to L5, and the mean values of T1 and T2 values were 1244.6 and 71.8 ms, respectively. (D) Photomicrograph (400× magnification; HE and PAS stain) showed the BMPC percentage was approximately 65%. BMPC, bone marrow plasma cell; HE, hematoxylin eosin; MM, multiple myeloma; PAS, periodic acid schiff; PR, partial response; ROI, area of interest.

For focal lesions, ROIs were placed on all myeloma lesions with diameter ≥5 mm in the lumbar spine, along the border of the lesions.^21^, ^22^ For non‐focal lesion (normal, salt‐and‐pepper, diffuse, combined diffuse and focal), the center region of the vertebral body from L1 to L5 was outlined as ROI to reduce the effect of cerebrospinal fluid and vertebral end‐plate changes (Figures 1B,C and 2B,C).^21^, ^22^ Data for each participant were expressed as the mean value of all ROIs.

### 2.5 Statistical analysis

The normality of the distributions of clinical and MRI variables was assessed by Shapiro–Wilk test. For univariate analysis, the clinical and MRI variables were compared between two groups using the independent *t*‐test (normal distribution) or Mann–Whitney *U*‐test (non normal distribution) for continuous variables and Chi‐square tests or Fisher's exact tests for categorical variables. Variables with a *p* < 0.05 in univariate analyses were included as covariates in multivariate (logistic regression) analyses. Predicting performance was assessed using area under the curve (AUC) of the receiver operating characteristic (ROC) curve. Comparison of SyMRI parameters between genders were performed by the Mann–Whitney *U*‐test. The distribution of participants based on SyMRI parameters between two groups at each R‐ISS stage was analyzed by Fisher's exact tests. Statistical significance was defined as *p* < 0.05. All statistical analyses were performed by using software (SPSS version 22.0; SPSS, Chicago, Ш).

## 3 RESULTS

### 3.1 Participant cohort

A total of 69 participants were initially included in this study. Three participants had other diseases in the lumbar spine, six participants had not completed the 4 cycles of induction chemotherapy in our hospital, and one participant had poor imaging quality. Ultimately, we collected 59 participants, including 11 in R‐ISS I stage, 36 in R‐ISS II stage, and 12 in R‐ISS III stage (Table 4). Among them 33 participants received good response and 26 received poor response after 4 cycles of induction chemotherapy (Table 1).

**TABLE 1:** Participant characteristics and group differences.

Characteristics	Participants with good response (*n* = 33)	Participants with poor response (*n* = 26)	*p*‐value
Gender			
Male	23	16	0.511^a^
Female	10	10	
Age (year)	62.3 ± 8.8	64.6 ± 7.4	0.294^b^
Hemoglobin (g/L)	100.6 ± 28.7	92.5 ± 24.3	0.258^b^
Platelet counts (10^9^/L)	172.0 (105.5, 262.5)	165.0 (81.8, 229.5)	0.663^c^
β_2_‐microglobulin (mg/L)	3.41 (2.73, 7.02)	6.78 (3.88, 10.92)	0.009^c^
Albumin (g/L)	34.4 (29.2, 40.2)	31.7 (27.6, 36.6)	0.174^c^
LDH (U/L)	178.0 (135.5, 228.0)	183.5 (137.0, 229.25)	0.982^c^
Calcium (mmol/L)	2.32 (2.18, 2.42)	2.28 (2.17, 2.77)	0.831^c^
Creatinine (umol/L)	78.0 (57.5, 103.0)	99.5 (65.5, 250.8)	0.051^c^
BMPC percentage (%)	27.4 ± 2.9	40.4 ± 4.4	0.013^b^
SyMRI parameters			
T1 value (ms)	755.8 (646.2, 1028.3)	1168.4 (1013.1, 1631.4)	<0.001^c^
T2 value (ms)	92.2 ± 14.1	76.4 ± 15.9	<0.001^b^
PD value (pu)	76.3 (69.1, 85.1)	76.9 (67.9, 82.9)	0.819^c^

*Note*: Data expressed as mean ± standard deviations for normally distributed data, or as median (interquartile range) for non‐normally distributed data.

Abbreviations: LDH, Lactate dehydrogenase; BMPC, bone marrow plasma cell.

^a^ The statistical method used chi‐square test.

^b^ The statistical method used independent *t*‐test.

^c^ The statistical method used Mann–Whitney *U*‐tests.

### 3.2 Comparison of clinical and MRI characteristics between two groups

Baseline characteristics for the two groups were shown in Table 1. Poor response group and good response group had similar clinical and MRI characteristics at diagnosis, except for higher BMPC percentage in poor response group (40.4% vs. 27.4%; *p* = 0.013), higher β_2_‐microglobulin in poor response group (6.78 mg/L vs. 3.41 mg/L; *p* = 0.009), higher T1 value in poor response group (1168.4 ms vs. 755.8 ms; *p* < 0.001), and lower T2 value in poor response group (76.4 ms vs. 92.2 ms; *p* < 0.001) when compared to good response group (Table 1; Figures 1 and 2).

### 3.3 Regression analyses for the prediction of poor response

Logistic regression analyses indicated that T1 value (odds ratio, 1.003; 95% CI, 1.001–1.005; *p* = 0.005) and T2 value (odds ratio, 0.910; 95% CI, 0.857–0.967; *p* = 0.002) (Table 2) were correlated with poor response, but BMPC percentage and β_2_‐microglobulin had no correlation with the poor response (Table 2).

**TABLE 2:** OR of poor response.

Variables	OR (95%CI)	*p‐*value
β_2_‐microglobulin	1.225 (0.994–1.511)	0.057
BMPC percentage	1.060 (0.023–49.491)	0.976
T1 value	1.003 (1.001–1.005)	0.005
T2 value	0.910 (0.857–0.967)	0.002

Abbreviations: CI, confidence interval; OR, odds ratio.

### 3.4 Comparison of SyMRI parameters between genders

There were no significant differences in SyMRI parameters between genders (Table 3).

**TABLE 3:** Comparison of SyMRI parameters between genders.

SyMRI parameters	Male	Female	*p*‐value
T1 value (ms)	944.0 (731.2, 1288.6)	1014.3 (549.4, 2144.0)	0.737
T2 value (ms)	81.4 (72.5, 95.0)	84.7 (76.7, 99.3)	0.496
PD value (pu)	77.8 (70.3, 83.5)	73.2 (67.4, 84.6)	0.317

*Note*: Data expressed as median (interquartile range) for non‐normally distributed data; the statistical method used Mann–Whitney *U*‐tests.

### 3.5 Diagnostic performance for prediction of early treatment response

**FIGURE 3:**
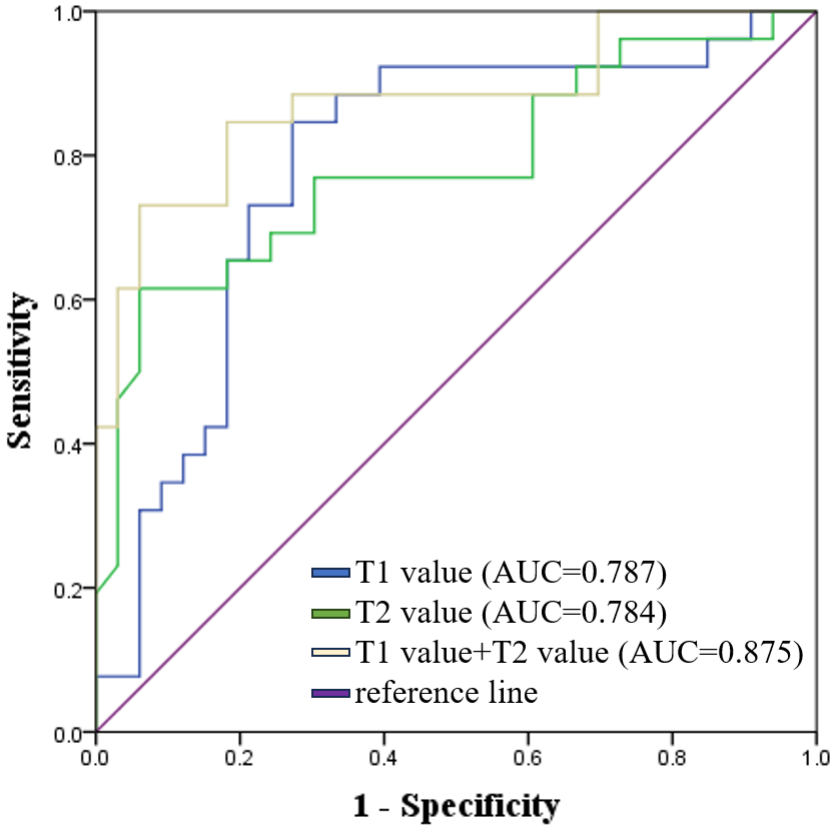
The ROC curves of T1 value, T2 value and a combination of these two factors for the diagnosis of poor early treatment response. AUC, area under the curve; ROC, receiver operating characteristic.

In ROC analysis, the T1 value (cutoff value of 967.2 ms) had sensitivity of 85.0%, specificity of 73.0%, and AUC of 0.787; and T2 value (cutoff value of 75.9 ms) demonstrated sensitivity of 94%, specificity of 61.0%, and AUC of 0.784 (Figure 3). The combination of T1 value and T2 value achieved sensitivity of 73.0%; specificity of 94.0%, and AUC of 0.875 (Figure 3).

### 3.6 Application of SyMRI parameters in R‐ISS stage

In R‐ISS II stage, there was a significant difference in the distribution of participant with different SyMRI parameters between the two groups (*p* < 0.001) (Table 4), while there was no significant difference in stage I and stage III (Table 4). In R‐ISS II stage, participants with neither T1 value ≥967.2 ms nor T2 value ≤75.9 ms were more likely to get good response, and participants with both T1 value ≥967.2 ms and T2 value ≤75.9 ms were potentially to get poor response (Table 4).

**TABLE 4:** Number of participants expressing different SyMRI parameters between two groups in each R‐ISS stage.

R‐ISS stage	Participants with good response (*n* = 33)	Participants with poor response (*n* = 26)	*p‐*value
R‐ISS I			
a	7	1	0.491
b	2	1	
c	0	0	
R‐ISS II			
a	16	1	<0.001
b	5	5	
c	1	8	
R‐ISS III			
a	1	1	0.227
b	1	3	
c	0	6	

*Note*: a, participants with neither T1 value ≥967.2 ms nor T2 value ≤75.9 ms; b, participants with T1 value ≥967.2 ms or T2 value ≤75.9 ms; c, participants with both T1 value ≥967.2 ms and T2 value ≤75.9 ms.

Abbreviation: R‐ISS, Revised International Staging System.

## 4 DISCUSSION

The quantitative parameters derived from SyMRI have been used for a variety of organs or diseases.^15^, ^16^, ^17^, ^18^, ^23^, ^24^, ^25^ Our study demonstrated that T1 and T2 values of lumbar marrow could predict the early treatment response in newly diagnosed MM and help to precise risk stratification of the R‐ISS II stage.

Our study indicated that higher BMPC percentage and higher β_2_‐microglobulin were associated with poor response. The BMPC percentage is critical for MM diagnosis, assessment of treatment efficacy, and monitoring for recurrence.^1^ The BMPC percentage was significantly correlated with tumor burden and prognosis in MM.^26^, ^27^, ^28^ Beta‐2‐microglobulin, a low‐molecular weight protein expressed on the surface of almost all normal nucleated cells and many tumor cells, is a major serum biomarker of tumor burden in MM and a component of R‐ISS staging system.^4^, ^7^, ^29^, ^30^ In addition, the serum β_2_‐microglobulin level was proved to be an independent predictor of survival in MM.^31^ However, our study showed that BMPC percentage and β_2_‐microglobulin were not independent predictors of early treatment response in our multivariate analysis. The reason may be that β_2_‐microglobulin is excreted by the kidneys and is inaccurate in renal failure, however, MM patients often have impaired renal function and BMPC percentage is susceptible to sampling error and spatial heterogeneity of disease.

Our study showed that higher T1 values and lower T2 values were associated with poor response in univariate analysis, and that T1 and T2 values were independent predictors of early treatment response in multivariate analysis. The AUC of T1 and T2 values were 0.787 and 0.784, respectively. The normal composition of BM in older adults is approximately 15% water, 80% fat, and 5% hematopoietic cells.^32^ The initiation and progression of MM is the result of the interaction between monoclonal plasma cells and the BM microenvironment, in which adipocytes can not only secrete various active ingredients to promote this process but also provide energy source for plasma cells proliferation.^33^ This can result in the decrease of fat content in the BM. In addition, monoclonal plasma cells proliferation will produce excessive immunoglobulin or light chains, which will increase the protein content in the BM. The T1 value is mainly related to the macromolecule concentration, and changes in both protein and fat content in MM may lead to changes in T1 values.^34^ The T2 value is mainly determined by the free water content which is small molecule and longer transverse relaxation.^35^ The presence of higher BMPC, nuclear polymorphism and the nucleo‐cytoplasmic ratios due to plasma cells proliferation, resulting in a corresponding reduction in the extracellular space and free water content.^36^ The study of Meng et al. showed that the T1 values of breast cancers were significantly higher than that of benign lesions, while the T2 values were lower than that of benign lesions.^15^ Zhao et al. analyzed 94 rectal cancer patients and found that T1 and T2 values were associated with different prognostic factors, respectively.^16^ Li et al. indicated that the T1 and T2 values in the hippocampal sclerosis area were significantly higher than those in the contralateral hippocampus and control group.^23^ Our results were consistent with previous studies, suggesting that T1 and T2 values may be potential markers for characterizing tissue composition and reflecting metabolic tumor burden in MM.

The PD value mainly reflects the water content.^34^ A previous study showed that the PD value could differentiate prostate cancer from glandular hyperplasia.^24^ Another study indicated that the PD value could distinguish between bone metastasis and posttreatment reactive sclerosis in prostate cancer.^17^ However, in our study, there was no significant difference in PD values between the two responder groups. The clinical application of PD value in MM needs to be further explored.

Previous studies have shown that BM fat is higher in older women than in men, MM usually occurs in older people, and BM fat plays an important role in the initiation and progression of the disease.^33^, ^37^ Our results showed that there were no significant differences in SyMRI parameters between genders. First, this may be due to the fact that the T1, T2 and PD values of SyMRI represent the transverse relaxation time, longitudinal relaxation time and proton density values of the tissue, respectively, which are the expression results of plasma cells proliferation and its metabolites interacting with the inherent substances of the bone marrow; second, it may be due to the small sample size in this study, with a total of 59 cases, including 39 male participants and 20 female participants.

The R‐ISS stage, which contains both gene mutation and tumor biological information, is now considered the most widely used risk stratification method. However, the efficacy of R‐ISS II stage needs to be improved because it is an exclusionary definition, with up to 62% of MM patients classified as II stage and their outcomes highly heterogeneous. In our study, participants with both T1 value ≥967.2 ms and T2 value ≤75.9 ms in R‐ISS II stage tend to receive poor response. So, T1 and T2 values based on metabolic tumor burden may serve as supplemental markers to improve risk stratification of R‐ISS II stage.

There were several limitations in the current study. First, it was a single‐center study with a small sample size. Large multicenter studies are needed to confirm these findings. Second, due to the long acquisition time of SyMRI, our study only applied it to lumbar marrow and only measured quantitative parameters at the central slice, and then the application of more anatomic sites and the use of artificial intelligence method to extract lesion features will be performed.

## 5 CONCLUSIONS

SyMRI‐derived T1 and T2 value were independent predictors of early treatment response to newly diagnosed MM, and supplementary markers that could promote R‐ISS II stage risk stratification. SyMRI may be a promising approach to evaluate the metabolic tumor burden of MM.

## AUTHOR CONTRIBUTIONS

**Sha Cui:** Conceptualization (lead); methodology (lead); software (equal); writing – original draft (lead). **Yinnan Guo:** Formal analysis (lead); methodology (equal); software (lead); visualization (lead). **Weiran Niu:** Software (equal). **Jianting Li:** Data curation (equal). **Wenjin Bian:** Software (equal). **Wenqi Wu:** Investigation (equal). **Wenjia Zhang:** Methodology (equal). **Qian Zheng:** Validation (equal). **Jun Wang:** Resources (equal). **Jinliang Niu:** Funding acquisition (lead); project administration (equal); resources (lead); supervision (lead).

## CONFLICT OF INTEREST STATEMENT

The authors have no conflict of interests to declare.

## FUNDING INFORMATION

This study has received funding by National Natural Science Foundation of China (NSFC 82071898).

## ETHICS STATEMENT

This prospective study received approval from the ethics committee of the Second Hospital of Shanxi Medical University (protocol number 2020–040). All participants provided written informed consent for the performance of MRI examination and for the use of clinical, laboratory, and imaging data.

## Data Availability

The data that support the findings of this study are available from the corresponding author upon reasonable request.
